# Icaritin-Induced FAM99A Affects GLUT1-Mediated Glycolysis *via* Regulating the JAK2/STAT3 Pathway in Hepatocellular Carcinoma

**DOI:** 10.3389/fonc.2021.740557

**Published:** 2021-10-26

**Authors:** Xia Zheng, Yudong Gou, Ziyu Jiang, Aizhen Yang, Zhihui Yang, Shukui Qin

**Affiliations:** ^1^ Nanjing University of Chinese Medicine, Nanjing, China; ^2^ Oncology Department, Jiangsu Province Hospital of Chinese Medicine, Nanjing, China; ^3^ Oncology Department, Affiliated Hospital of Integrated Traditional Chinese and Western Medicine, Nanjing University of Chinese Medicine, Nanjing, China; ^4^ Laboratory Department, Nanjing Jinling Hospital, Nanjing, China; ^5^ Pathology Department, Nanjing Jinling Hospital, Nanjing, China; ^6^ Oncology Department, Nanjing Jinling Hospital, Nanjing, China

**Keywords:** icaritin, FAM99A, glycolysis, GLUT1, hepatocellular carcinoma

## Abstract

Icaritin is a potential treatment option for hepatocellular carcinoma (HCC) based on the results of its phase 2 stage trial. Glucose transporter 1 (GLUT1), a critical gene in regulating glycolysis, has been recognized as a promising target in HCC treatment. Previous studies have reported that FAM99A, a new long noncoding (lncRNA), is associated with HCC metastasis. It has also been demonstrated that the JAK2/STAT3 pathway is related to HCC and is the target of icaritin treatment. However, whether FAM99A participates in icaritin treatment and regulates GLUT1-mediated glycolysis *via* the JAK2/STAT3 pathway in HCC cells remains to be explored. Our study aimed to clarify the mechanisms underlying glycolysis and understand the regulating effects of the FAM99A and JAK2/STAT3 pathway in HCC cells in icaritin treatment. Molecular mechanism studies were conducted to verify whether FAM99A could bind to the JAK2/STAT3 pathway and to identify the regulatory mechanisms in the HCC cells. It was revealed that icaritin inhibited proliferation, GLUT1 level, and the glycolysis of the HCC cells. FAM99A in HCC cells was upregulated after a high concentration treatment of icaritin. FAM99A inhibited GLUT1 by blocking the JAK2/STAT3 pathway. Mechanically, FAM99A interacted with EIF4B to inhibit gp130 and gp80 translation, which then interacted with miR-299-5p to upregulate SOCS3, causing the JAK2 pathway to inhibit STAT3 phosphorylation, so that JAK2/STAT3 was blocked in HCC cells. Overall, our study proved that icaritin-induced FAM99A can inhibit HCC cell viability and GLUT1-mediated glycolysis *via* blocking the JAK2/STAT3 pathway.

## Introduction

Hepatocellular carcinoma (HCC) is the fifth most common malignancy and the third leading cause of cancer-related death globally (1). The incidence of HCC and HCC-related deaths has increased over the last several decades (2). Aerobic glycolysis, one of the hallmarks of cancer, is a typical energy metabolism reprogramming process and contributes to the malignant biological properties of cancers, including HCC (3, 4). Glucose transporter 1 (GLUT1), commonly overexpressed in multiple tumor tissues, promotes glycolysis through increasing glucose intake in cancer cells and is considered a promising therapeutic target in HCC treatment (5). Icaritin is a natural product isolated from plants of the genus *Epimedium* and has been tested in human patients. Based on the result of a phase 2 stage clinical trail (6), we conducted a phase 3 clinical study for icaritin to treat advanced HCC (NCT03236649). However, the mechanisms underlying the effect of icaritin has not been clarified definitively.

Long noncoding RNAs (lncRNAs) have been proven to be associated with diverse fundamental biological processes (7), and some of them have been confirmed to be involved in the progression of HCC cells. For example, in HCC, lncRNA DPIA3P1 regulates chemoresistance by nuclear factor (NF)-kappa B signaling pathway (8). Also, lncRNA HAND2-AS1 enhances the stemness of HCC cells (9). lncRNA PSTAR facilitates P53 signaling to suppress HCC (10). A newly found lncRNA, FAM99A, has been reported to be related to hypoxia-induced HCC metastasis (11). However, if and how FAM99A affects the proliferation and glycolysis of HCC cells has not yet been studied.

The activation of the JAK2/STAT3 pathway, as a known target of icaritin, has been reported to play critical roles in several solid tumors. For example, the JAK2/STAT3 pathway is prominent in the progression of ovarian cancer (12), and it has been proven to be associated with metastasis in breast cancer (13). As for the relationship between HCC cells and JAK2/STAT3 pathway, previous studies have demonstrated that interleukin 7 (IL-17) can induce the JAK2/STAT3 signaling pathway *via* AKT1 to aggravate HCC (14). Moreover, emerging research indicates that lncRNAs play a vital role in regulating the JAK2/STAT3 pathway in cancers. For example, lnc-BM promotes the JAK2/STAT3 pathway to facilitate brain metastasis in breast cancer (15). Furthermore, DLGAP1 contributes to tumorigenesis in HCC *via* the JAK2/STAT3 pathway (16). However, if and how FAM99A regulates the JAK2/STAT3 pathway in HCC cells is still unclear. Moreover, although icaritin has been confirmed to be an anticancer agent involved in activating the JAK2/STAT3 pathway (17), the specific mechanism behind icaritin regulating the JAK2/STAT3 pathway is not well understood.

Therefore, in this study, we attempted to determine whether icaritin could induce FAM99A to regulate the JAK2/STAT3 pathway and affect GLUT1-mediated glycolysis in HCC cells. We also further investigated the mechanisms of how this pathway is affected.

## Materials and Methods

### Cell Culture

HepG2 and HCCLM3 cells were purchased from the Cell Bank of the Chinese Academy of Sciences (Shanghai, China). Cells were grown in Dulbecco’s Modified Eagle Medium (DMEM; HyClone, South Logan, UT, USA) with 10% fetal bovine serum (FBS; PAN-Biotech, Aidenbach, Germany) under 5% CO_2_ at 37°C.

### Cell Transfection

Lipofectamine 2000 Reagent (Invitrogen, Carlsbad, CA, USA) was applied for the cell transfection. HepG2 or HCCLM3 cells were added to six-well plates until cell confluence was nearly at 80%. The pcDNA3.1 vector and the targeting FAM99A and STAT3 genes were synthesized by GenePharma (Shanghai, China). Small interfering RNAs (siRNAs; si-FAM99A) and si-NC (negative control) were purchased to silence the related genes. Lentivirus (Lv; Lv-FAM99A) and its Lv-NC were procured and used in this study. For the overexpression of microRNA (miR)-299-5p, miR-299-5p-mimics or NC mimics were cotransfected with FAM99A-WT or FAM99A-MUT into HepG2 cells.

### Quantitative Real-Time Polymerase Chain Reaction

In line with the instruction of the TRIzol reagent (Takara, Japan), the total RNA samples were extracted from HepG2 and HCCLM3 cells. Synthesis of complement DNA (cDNA) for miR-299-5p was carried out using the TaqMan MicroRNA Reverse Transcription Kit (Takara, Japan). The cDNA for FAM99A was synthesized by the PrimeScript RT reagent Kit (Takara, Japan). The quantitative real-time polymerase chain reaction (qRT-PCR) reaction was conducted using the SYBR Green PCR Kit (Sigma-Aldrich, St. Louis, MO, USA) and followed by the 2^−ΔΔCt^ method. In relevant assays, dimethyl sulfoxide (DMSO) or the control was used as the loading control.

### MTT Assay

After transfection or treatment with icaritin, the cell viability of the HepG2 cells under different conditions was observed in 96-well plates, in line with the methods of the MTT assay. Absorption was measured with the microplate reader at 490 nm.

### Colony Formation Assay

HCC cells were plated in six-well plates with 6 × 10^2^ cells in each well in triplicate. After that, cells were incubated for 1 day at 37°C. After cells were removed from the culture medium, a fresh culture medium with different doses of icaritin was added to each well, which was followed by 48 h of incubation at 37°C. Then, a special culture medium was substituted with a culture medium without icaritin. Colonies were observed under a microscope, and a digital camera captured the photos of plates. Colonies with >50 cells were evaluated.

### EdU Incorporation Assay

HCC cells treated with different doses of icaritin were cultivated with an EdU incorporation assay kit (100 ml, RiboBio, Guangzhou, China). Then, 4% paraformaldehyde was used for fixation. Following permeabilization, cell nuclei were stained by DAPI. The proliferative HCC cells were determined visually by a fluorescent microscope (Leica, Wetzlar, Germany).

### Seahorse Assay

We utilized the Seahorse XF 96 Extracellular Flux Analyzer (Agilent) to determine the extracellular acidification rate (ECAR). According to the manufacturer’s instructions, ECAR was examined with a Seahorse XF glycolysis stress test kit. Briefly, 2 × 10^4^ HepG2 or HCCLM3 cells per well were treated with various concentrations of icaritin for 48 h and seeded into a Seahorse XF 96 cell culture plate with 15% fetal bovine serum DMEM overnight. Then, cells were washed and incubated with base medium with 2 mM L-glutamine for 1 h at a 37°C, CO_2_-free incubator. After three baseline measurements, glucose, oligomycin, and 2-DG were sequentially added into each well at the time points specified to a final concentration of 10 mM, 10 μM, or 50 mM, respectively. ECAR dates were assessed by Seahorse XF 96 Wave software.

### Glucose Consumption and Lactate Production

2-NBDG (Life Technologies) was used as a glucose tracer. Briefly, the pre-treated cells were seeded in six-well plates with 1 × 10^5^ cells per well and incubated overnight at 37°C with 5% CO_2_. The next day, cells were cultured with glucose-free condition for 4 h, and then one well was incubated with DMEM medium with 25 μM glucose. As a negative control group, cells were cultivated with 25 μM 2-NBDG for 2 h. After incubation, cells were digested and washed twice with PBS. We detected the mean fluorescence intensity of cells by flow cytometry with excitation light at 488 nm.

To measure lactate production, 1 × 10^5^ cells per well were seeded in 24-well plates in triplicate for 24 h, and then the medium was refreshed with DMEM containing 1 mM glucose overnight. The next day, culture medium was collected for measurement of lactate as determined by the lactate assay kit (Biovision). Lactate production was adjusted by cell numbers.

### Western Blot Assay

Extraction of the total protein was performed by using RIPA (Thermo Fisher Scientific, Waltham, MA, USA). Protein was separated with 10% SDS-PAGE (Bio-Rad Laboratories, Hercules, CA, USA). Next, the protein was transferred onto polyvinylidene fluoride (PVDF) membranes (Millipore, Billerica, MA, USA). The membrane was then incubated with the primary antibodies. Antibodies included anti-GLUT1 (ab115730), anti-STAT3 (ab32500), anti-p-STAT3 (ab76315), anti-JAK2 (ab108596), anti-p-JAK2 (ab32101), anti-EIF4B (ab245618), anti-gp80 (ab222101), and anti-beta Actin (ab8226), all of which were acquired from Abcam (Cambridge, UK); anti-gp130 (#3732), which was acquired from Cell Signaling Technology (CST; Danvers, MA, USA), was also used. Next, the membrane was incubated with secondary antibodies. The relative expression level of the protein was assessed using Image J software (NIH Image, Bethesda, MD, USA).

### Luciferase Reporter Assay

For luciferase reporter assay, FAM99A-WT/MUT or SOCS3-WT/MUT was subcloned into the pmirGLO luciferase reporter vector (GeneChem). The Dual-Luciferase Reporter Assay System (Promega, Madison, WI, USA) was then applied according to the manufacturer’s instructions.

### RNA Pull-Down Assay

Biotinylated FAM99A (1 μg) was taken and then treated with a structured buffer to form a secondary structure. The secondary structure was then heated with FAM99A at 95°C for 2 min. After that, it was put into an ice bath for 3 min and statically treated for 30 min. After this, 15 μl of streptavidin beads was added to the biotin-labeled and denatured FAM99A and spun at 4°C for 2 h. The target sequences were synthesized and biotinylated into the probe with a cell lysis solution. The number of cells was 1–2 × 10^7^. Cell protein extracts were then prepared to be mixed with magnetic beads, Bio-sense, and Bio-antisense. After that, the samples were rotated and incubated overnight at 4°C. Centrifugation was then carried out after incubation, and RNA immunoprecipitation **(**RIP) wash buffer (40 μl) and 5× SDS loading buffer (10 μl) were added to the samples at 95°C for a 10-min vibration (1,000 rpm). Following this, electrophoresis and silver staining were conducted using the PAGE Gel Silver Stain Kit. Mass spectrometric analysis was then conducted based on differences between the silver staining bands.

### RIP Assay

The Magna RIP Kit (Millipore) was used for performing the RIP assay. The HepG2 cells were lysed in the RIP lysis buffer, and then cell extracts were treated with the RIP buffer using magnetic beads conjugated with anti-JAK2 (ab108596, Abcam) or anti-immunoglobin G (IgG; ab172730, Abcam). The precipitated RNAs were then analyzed by qRT-PCR.

### *In Vivo* Tumor Growth Assay

To determine tumor growth, 1 × 10^6^ HepG2 cells were transfected for 48 h and injected subcutaneously into female nude mice, with tumor growth being subsequently monitored every 4 days. After the mice were killed, the excised tumors were measured for analysis.

### Statistical Analysis

Data from at least three independent experiments are presented as the mean ± standard deviation. Prism 6.0 (GraphPad, San Diego, CA, USA) was used for all analyses. A two-tailed Student’s *t*-test or one-way analysis of variance (ANOVA) was used for analyzing differences. A *p*-value <0.05 was considered the threshold for statistical significance.

## Results

### Icaritin Reduced the Proliferation of HCC Cells

**Figure 1:**
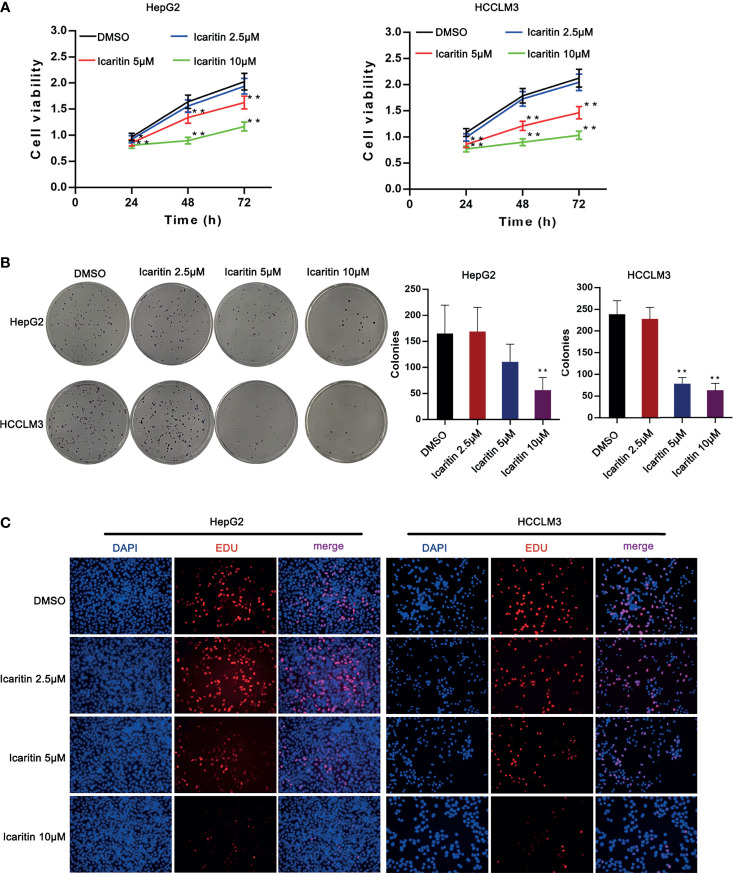
Icaritin affected the proliferation of HCC cells. **(A)** HCC cell viability was detected after the treatment of icaritin of different concentrations by MTT assay. **(B)** Pictures of colonies and quantification of colony number generated by HCC cells under the treatment of different doses of icaritin. **(C)** Pictures of EdU-stained proliferative HCC cells under the treatment of different doses of icaritin. ^*^ *p* < 0.05, ^**^ *p* < 0.01.

According to previous studies, two cell lines (HepG2 and HCCLM3) were selected for our study. To verify the biological characteristics, we used DMSO to treat the HCC cells, as it has a very strong affinity for water and exposure to air and can be rapidly diluted (18). Moreover, icaritin of different concentrations was used to treat HepG2 and HCCLM3 cells, as it was proven to be associated with the progression of HCC cells. Therefore, we first detected the influence of icaritin on the proliferation of HCC cells. As shown in **Figure 1A**, the cell viability of HepG2 and HCCLM3 cells decreased as the dose of icaritin increased from 2.5 to 10 μm. Icaritin reduced colonies generated by HCC cells in a dose-dependent manner (**Figure 1B**). EdU-stained proliferative HCC cells were reduced as the treatment dose of icaritin increased (**Figure 1C**). To conclude, icaritin attenuates HCC cell proliferation.

### Icaritin Reduced GLUT1 Expression and Inhibited the Warburg Effect in HCC Cells

**Figure 2:**
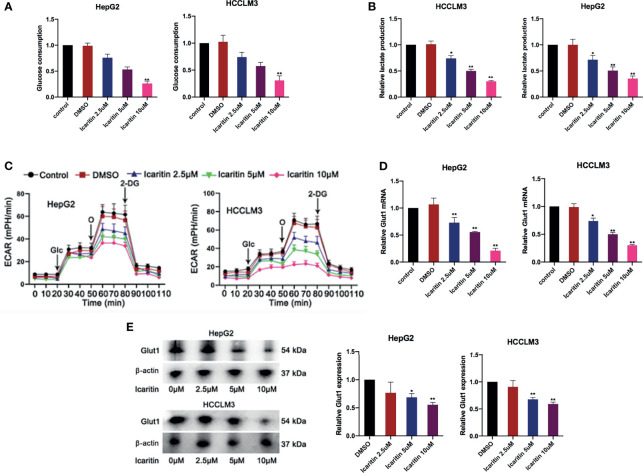
Icaritin reduced GLUT1 and moderated the Warburg effect in HCC cells. **(A, B)** Glucose consumption and lactate production efficiency of HCC cells were detected after the treatment of icaritin of different concentrations. **(C)** ECAR of HCC cells with the treatments of different concentrations icaritin. **(D, E)** The mRNA and protein level of GLUT1 in HCC cells was detected after the treatment of different concentrations icaritin. ^*^ *p* < 0.05, ^**^ *p* < 0.01.

We then explored the potential target of icaritin in HCC. Interestingly, we firstly demonstrated that the glucose consumption and lactate production in the HCC cells showed an icaritin dose-dependent reduction in HepG2 and HCCLM3 cells (**Figures 2A, B**). In addition, it revealed that the reduction of extracellular acidification rate (ECAR) was also performed in an icaritin dose-dependent manner (**Figure 2C**). A previous study determined that GLUT1 is a typical glycolytic gene (19), and the Warburg effect is prevalent in human cancer. Accordingly, most cancer cells display highly elevated glycolysis (20). Thus, we detected whether icaritin regulated GULT1 expression and the Warburg effect in HCC cells. First, GLUT1 messenger RNA (mRNA) and protein expression was validated to show a gradual decrease as the icaritin dose increased in HCC cells (**Figures 2D, E**). In summary, icaritin reduces GLUT1 expression and inhibits the Warburg effect in HCC cells.

### Icaritin Reduced GLUT1 Through Upregulating FAM99A in HCC Cells

**Figure 3:**
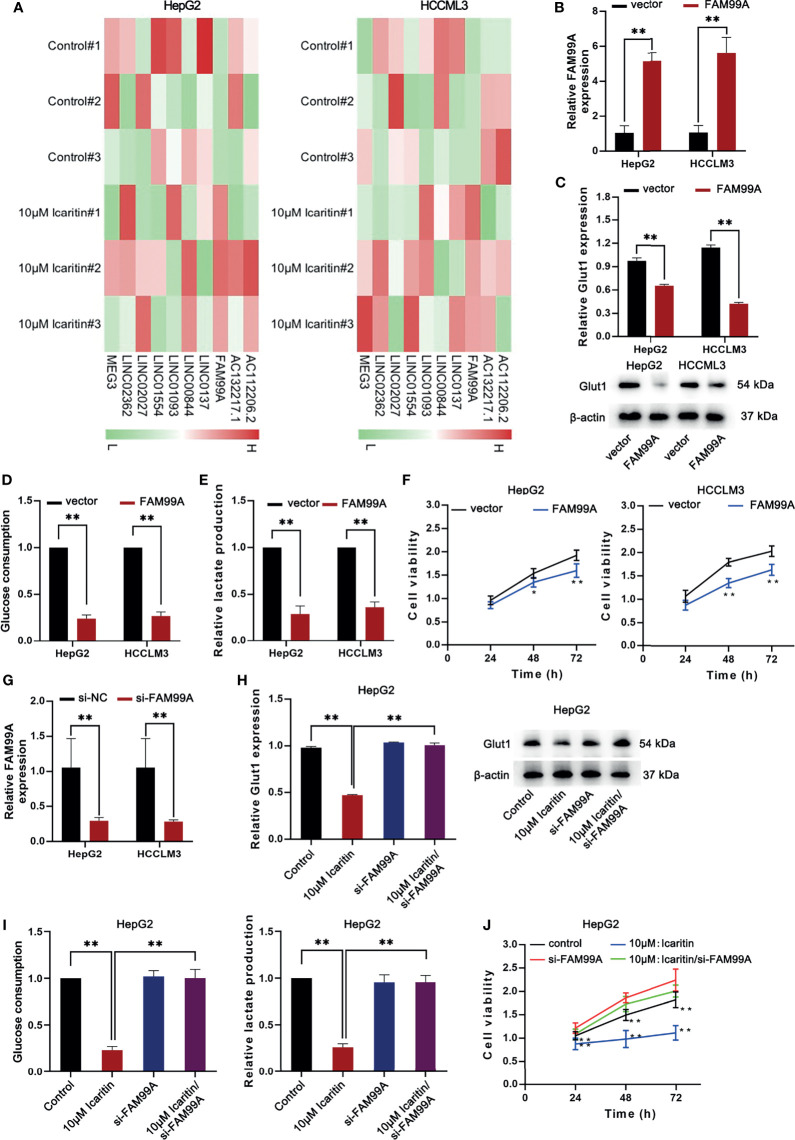
Icaritin moderated the proliferation and Warburg effect of HCC cells *via* regulating the expression of FAM99A. **(A)** The heatmap shows the transcriptome sequencing of differentially expressed lncRNAs in HCC cells treated with icaritin (10 μm). **(B)** The expression of FAM99A was detected by qRT-PCR after HepG2 and HCCLM3 cells were transfected with pcDNA3.1/FAM99A. **(C)** The GLUT1 mRNA expression and protein levels in HCC cells were detected after the overexpression of FAM99A by qRT-PCR and Western blotting assays. **(D, E)** Glucose consumption and lactate production of HepG2 and HCCLM3 cells were detected. **(F)** MTT assay showed that the overexpression of FAM99A could lead to decreased cell viability of HCC cells. **(G)** The expression of FAM99A was detected. **(H–J)** Cell viability, GLUT1 expression, glucose consumption and lactate production of HCC cells transfected with si-FAM99A were detected. *P < 0.05, **P < 0.01.

Subsequently, we investigated how icaritin regulated GLUT1 expression. Recently, lncRNAs have been demonstrated to be substantial gene regulators in HCC (21, 22); therefore, we hypothesized whether certain lncRNAs influence icaritin on GLUT1 expression. In previous studies (23), a transcriptome sequencing heatmap of the differentially expressed lncRNAs in HCC cells treated with 10 μM icaritin was generated (**Figure 3A**). As the expression of FAM99A was upregulated most significantly with icaritin treatment in HCC cells, we suspected that FAM99A mediated the effects of icaritin on GLUT1.

Subsequently, we sought to determine the effects of FAM99A on GLUT1 level in HCC cell lines. As shown in **Figure 3B**, FAM99A expression was upregulated after the HepG2 and HCCLM3 cells were transfected with pcDNA3.1/FAM99A. We verified that GLUT1 mRNA and protein levels dropped under FAM99A overexpression in HCC cells (**Figure 3C**). Glucose consumption and lactate production were reduced by FAM99A overexpression (**Figures 3D, E**). The viability of the HCC cells was reduced under FAM99A overexpression (**Figure 3F**). After that, the interference efficiency of FAM99A in HepG2 and HCCLM3 cells transfected with si-FAM99A was confirmed by qRT-PCR (**Figure 3G**). We found that GLUT1 mRNA and the proteins were reduced by icaritin treatment, and they recovered with FAM99A knockdown (**Figure 3H**). FAM99A knockdown countervailed the suppressive effect of icaritin on glucose consumption and lactate production (**Figure 3I**). Similarly, the downregulation of FAM99A recovered the viability of the HCC cells reduced under 10 μM icaritin treatment (**Figure 3J**). To conclude, icaritin reduces GLUT1 through upregulating FAM99A in HCC cells.

### FAM99A Moderated the Warburg Effect Through the JAK2/STAT3 Pathway

**Figure 4:**
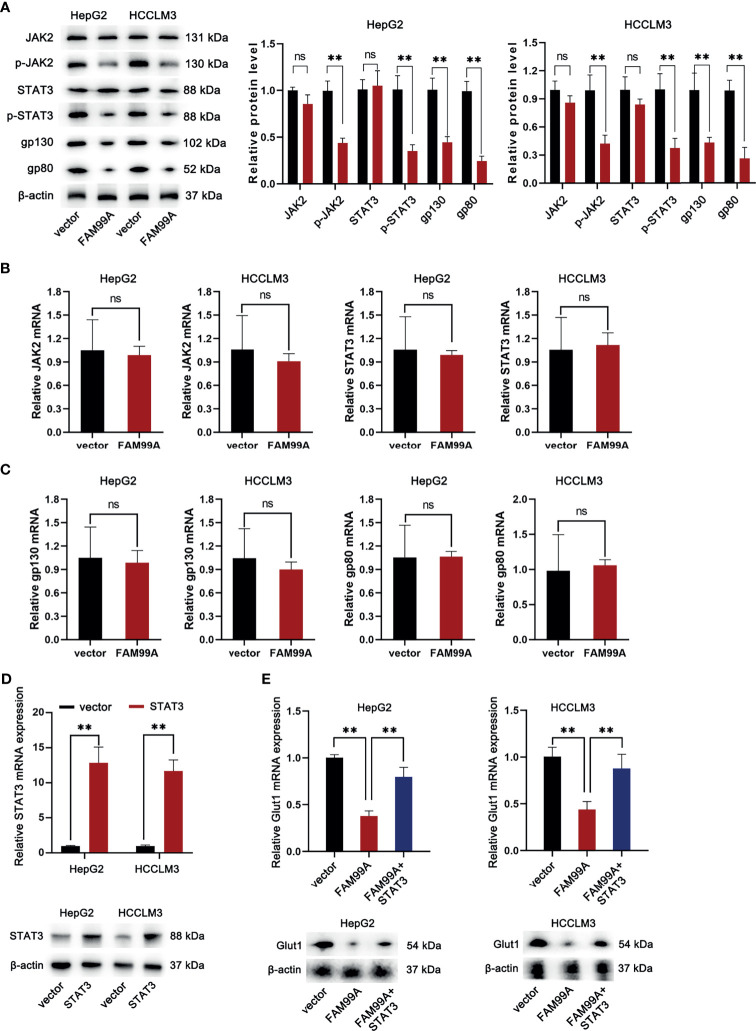
FAM99A moderated the Warburg effect through the JAK2/STAT3 pathway. **(A–C)** The mRNA expression of JAK2 and STAT3, gp130, and gp80, and protein level of JAK2/p-JAK2, STAT3/p-STAT3, gp130, and gp80 in HCC cells were detected after the overexpression of FAM99A. **(D)** The mRNA expression and protein level of STAT3 in HCC cells were detected by qRT-PCR and Western blotting assays. **(E)** GLUT1 expression was detected by qRT-PCR and Western blotting assays after the HCC cells were transfected with vector pcDNA3.1/fam99a or pcDNA3.1/FAM99A+pcDNA3.1/STAT3. ^**^ *p* < 0.01, ns, no significance.

As we confirmed that icaritin can moderate the Warburg effect of HCC cells *via* enhancing the expression of FAM99A, the following assays were conducted to verify whether FAM99A influences the Warburg effect through the JAK2/STAT3 pathway. First, the mRNA expressions of JAK2 and STAT3 and the protein levels of JAK2/p-JAK2 and STAT3/p-STAT3 in HCC cells were detected after FAM99A overexpression (**Figures 4A, B**).The overexpression of FAM99A was found to cause no obvious changes in the mRNA levels of JAK2 and STAT3 in HCC cells compared with the control groups. However, the protein levels of phosphorylated JAK2 and STAT3 (p-JAK2 and p-STAT3) were decreased by FAM99A overexpression. Previous studies have identified two transmembrane glycoproteins (the ligand-binding gp80 and the signal-transducer gp130) that form the functional IL-6 receptor (24, 25). Here, we found that the protein levels, rather than the mRNA levels of gp130 and gp80 in HCC cells, dropped significantly after FAM99A overexpression (**Figures 4A, C**). Next, we sought to identify whether FAM99A regulated GLUT1 through the JAK/STAT3 pathway. We observed an increase in mRNA and protein levels of STAT3 in HCC cells transfected with pcDNA3.1/STAT3 according to qRT-PCR and Western blotting assays (**Figure 4D**). The mRNA and protein levels of GLUT1 were detected by qRT-PCR and Western blotting assays after the HCC cells were transfected with pcDNA3.1/FAM99A+pcDNA3.1/STAT3 (**Figure 4E**). Our results showed that the decreases of mRNA and protein levels of GLUT1 under the overexpression of FAM99A were recovered by the transfection of pcDNA3.1/FAM99A+pcDNA3.1/STAT3. In summary, FAM99A can moderate the GLUT1-mediated Warburg effect through the JAK2/STAT3 pathway.

### FAM99A Blocked the Translation of gp130 and gp80 by Competitively Binding to EIF4B

**Figure 5:**
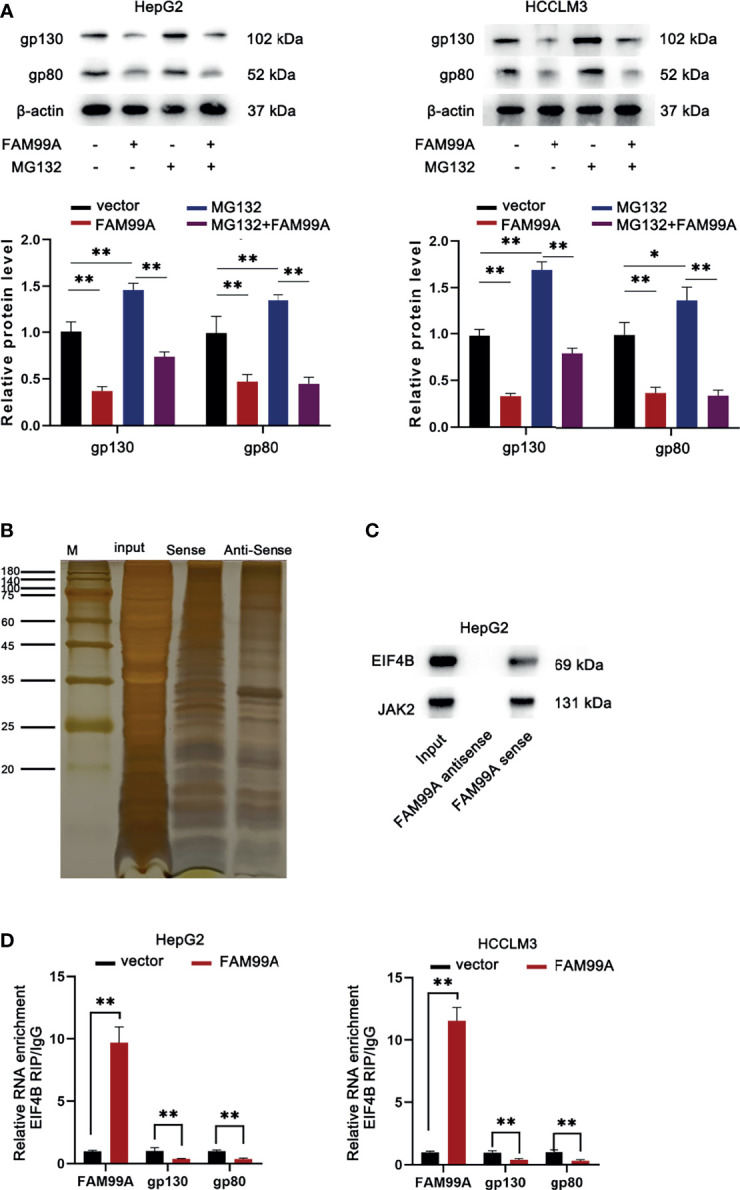
FAM99A blocked the translation of gp130 and gp80 *via* binding to EIF4B. **(A)** Western blotting assay was used to detect the protein levels of gp130 and gp80 in HCC cells after the transfection of pcDNA3.1/FAM99A and treatment of MG132. **(B)** Silver staining and differential band mass spectrometry assays were conducted to analyze the binding protein pulled down by FAM99A. **(C)** Western blotting verified the presence of EIF4B and JAK2 in the products pulled down by biotinylated FAM99A. **(D)** RIP and qRT-PCR assays were conducted to detect the enrichment of FAM99A, gp130, and gp80 in EIF4B precipitates in HepG2 cells. *P < 0.05, ^**^ *p* < 0.01.

As we surprisingly found that FAM99A overexpression could inhibit gp130 and gp80 protein levels but failed to alter their mRNA, we further probed how FAM99A regulated gp130 and gp80. First, we detected whether FAM99A regulated gp130 and gp80 at a posttranslational level. We found that, with or without the addition of proteasome inhibitor MG132 (26), FAM99A overexpression significantly reduced the level of gp130 and gp80 (**Figure 5A**), indicating that FAM99A regulated gp130 and gp80 at a translation level rather than a posttranslation level. Furthermore, silver staining and mass spectrometry assays of differential bands were conducted to analyze the binding protein pulled down by FAM99A and EIF4B was sorted out as a FAM99A-binding protein for subsequent studies (**Figure 5B**). A Western blotting assay was later adopted to verify the presence of EIF4B and JAK2 in the products pulled down by the biotinylated FAM99A (**Figure 5C**). According to previous studies, EIF4B is an important translation regulator in cancer (27) and can interact with lncRNA GMAN in HCC cells to promote its progression (28). Therefore, we suspected that EIF4B participated in the modulation of FAM99A on gp130 and gp80 translation. RIP and qRT-PCR assays were conducted to verify whether EIF4B could bind to FAM99A (**Figure 5D**). The result showed that FAM99A overexpression increased FAM99A enrichment and decreased gp130 and gp80 enrichment in EIF4B precipitates, indicating that FAM99A blocks the translation of gp130 and gp80 *via* binding to EIF4B.

### FAM99A Blocked the JAK2/STAT3 Pathway by Promoting SOCS3 Through Competing Endogenous RNA

**Figure 6:**
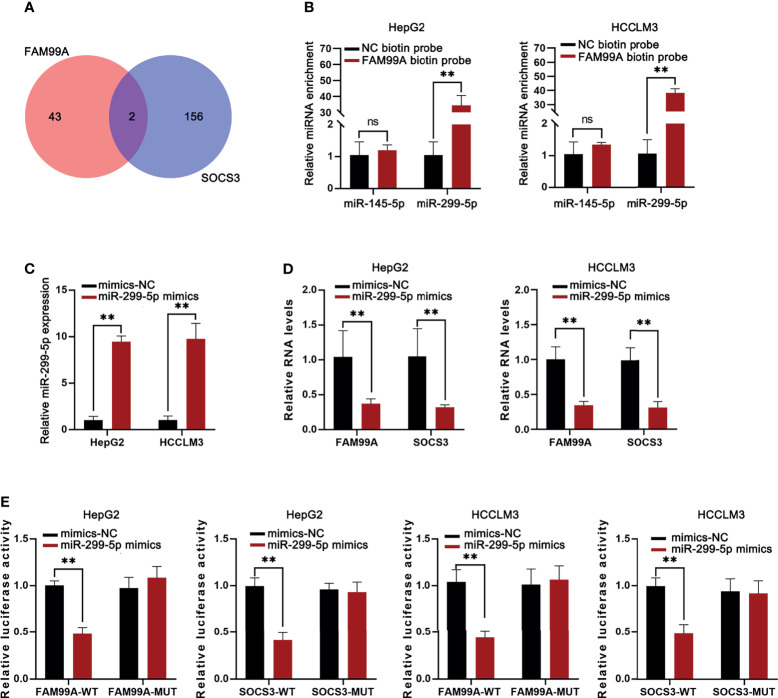
FAM99A blocked the JAK2/STAT3 pathway *via* promoting SOCS3. **(A)** miRNAs binding to both FAM99A and SOCS3 in HCC cells were sorted. **(B)** RNA pull-down assay was conducted to detect the enrichment of miR-145-5p and miR-299-5p in the pull-down of biotinylated FAM99A in HCC cells. **(C)** The overexpression efficiency of miR-299-5p was detected by qRT-PCR. **(D)** Expression of FAM99A and SOCS3 in HepG2 cells transfected with miR-299-5p mimics was detected. **(E)** Luciferase reporter assay was used to verify the relationship between FAM99A/SOCS3 and miR-299-5p. ^**^ *p* < 0.01, ns, no significance.

We identified that FAM99A failed to alter STAT3 and JAK2 expression but that it could alter phosphorylation levels and then further explored the underlying mechanisms. Previous studies have shown that SOCS3 is associated with the JAK2/STAT3 pathway in HCC cells (29). Therefore, we hypothesized that FAM99A regulates JAK2/STAT3 signaling through SOCS3. First, the manner in which miRNAs bind to FAM99A and SOCS3 in HCC cells was predicted (miR-145-5p and miR-299-5p; **Figure 6A**). Then, an RNA pull-down qRT-PCR assay revealed the enrichment of miR-299-5p instead of miR-145-5p in the pull-down of biotinylated FAM99A in HCC cells (**Figure 6B**). After that, the overexpression efficiency of miR-299-5p in HCC cells was detected by qRT-PCR (**Figure 6C**). We then detected FAM99A and SOCS3 expression in HCC cells transfected with miR-299-5p mimics and found that the overexpression of miR-299-5p led to a decrease in the expression of both FAM99A and SOCS3 (**Figure 6D**). Also, a luciferase reporter assay was adopted to verify the relationship between FAM99A/SOCS3 and miR-299-5p (**Figure 6E**). As shown by our results, both FAM99A and SOCS3 could bind to miR-299-5p. To conclude, FAM99A blocks the JAK2/STAT3 pathway *via* promoting SOCS3.

### FAM99A Inhibited STAT3 Phosphorylation to Affect the JAK2/STAT3 Activity *via* Binding to JAK2

**Figure 7:**
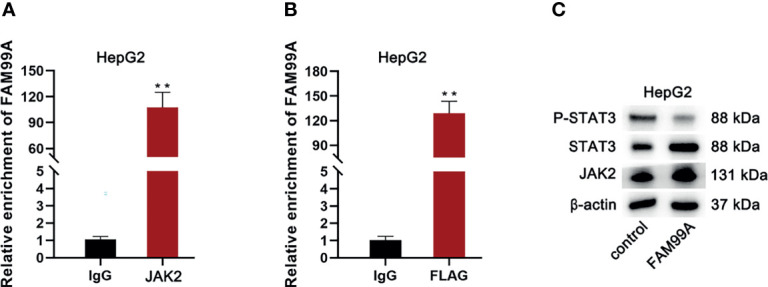
FAM99A inhibited STAT3 phosphorylation to affect the JAK2/STAT3 activity *via* binding to JAK2. **(A)** RIP and qRT-PCR assays were conducted to verify whether FAM99A could bind to JAK2. **(B)** We adopted RIP and qRT-PCR assays to verify whether FAM99A could bind to FLAG with the JH1 catalytic domain. **(C)** Western blotting assay was conducted to detect the effect of FAM99A on phosphorylated STAT3. ^**^ *p* < 0.01.

Subsequently, we attempted to determine whether there was an alternate way for FAM99A to regulate STAT3 phosphorylation. First, qRT-PCR and RIP assays were conducted to determine the relationship between FAM99A and JAK2 (JH1 catalytic domain; **Figure 7A**). As shown by our results, FAM99A could bind to JAK2. After HepG2 cells were cotransfected with a FLAG containing a JH1 catalytic domain and pcDNA3.1/FAM99A plasmids, we found that FLAG could bind to FAM99A (**Figure 7B**). Then, Western blotting tests showed that FAM99A overexpression did not impact JAK2 and STAT3 but did decrease phosphorylated STAT3 levels (**Figure 7C**). In summary, FAM99A inhibits STAT3 phosphorylation, impacting JAK2/STAT3 activity through JAK2 binding.

### The Effect of FAM99A on HCC in a Xenograft Model

**Figure 8:**
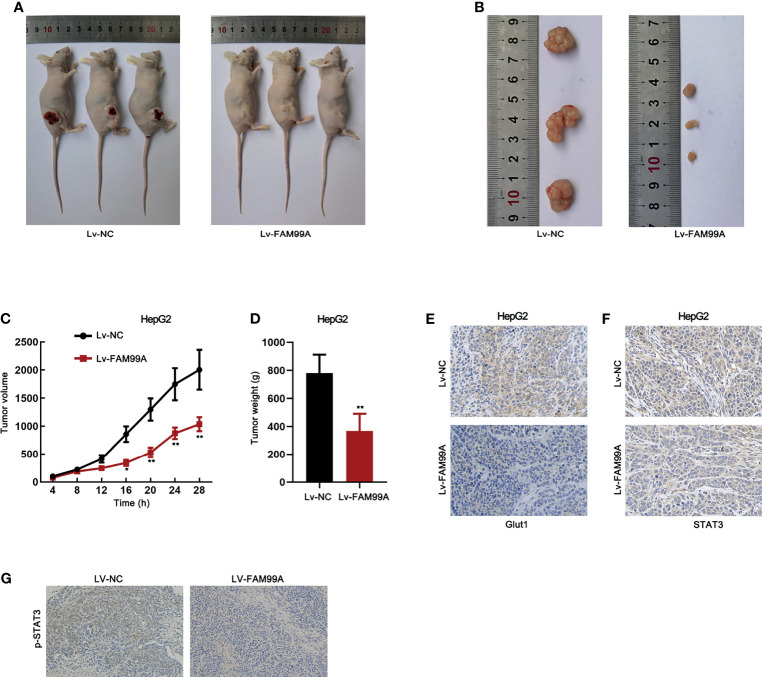
Effect of FAM99A on HCC in a xenograft model. **(A)** Pictures of mice with tumors generated by injected HepG2 cells transfected with Lv-FAM99A or lv-NC. **(B)** Pictures of dissected xenografts of each group. **(C)** The growth curve of xenografts was plotted by measuring the tumor volume in mice every 4 days after injection of transfected cells. **(D)** Tumor weight was evaluated after the tumors were dissected after 28 days. **(E)** The expression of GLUT1 in tumor tissues was detected by immunohistochemistry assay. **(F)** The expression and location of STAT3 in HepG2 was examined by immunohistochemistry assay. **(G)** The existence of p-STAT3 in tumor tissues was detected by immunohistochemistry assay. ^*^ *p* < 0.05, ^**^ *p* < 0.01.

To further confirm the *in vitro* data, we implemented animal experiments by establishing a xenograft model. HepG2 cells transfected with lv-NC or lv-FAM99A were injected into nude mice. The images of mice with tumors and the dissected tumors showed that xenografts in mice with lv-FAM99A were smaller than those in the control group (**Figures 8A, B**). Moreover, the growth curve revealed that xenografts grew slower in the mice of the lv-FAM99A group than in those of the control (**Figure 8C**). Tumor weight was reduced in the mice of the FAM99A overexpression group *versus* those in the control group (**Figure 8D**). Expression of GLUT1 was lowered in mice with FAM99A overexpression (**Figure 8E**). Also, it was verified that FAM99A overexpression caused the retention of STAT3 in the cytoplasm of HepG2 *in vivo* (**Figure 8F**). In addition, it was revealed that p-STAT3 level was decreased by FAM99A overexpression (**Figure 8G**).

## Discussion

Icaritin has been shown to be a potentially effective treatment for HCC in recent years (6). However, the mechanisms involved in how icaritin affects HCC have not been fully elucidated. Here, we used icaritin to treat the HCC cells and found that icaritin could reduce the proliferation of HCC cells. Interestingly, we found that glycolysis reactive and GLUT1 were reduced by icaritin in HCC cells. GLUT1 is a glycolytic gene while glycolysis is ubiquitous in cancer progression, including HCC (19, 20). We first determined that icaritin is a natural glycolysis inhibitor and is related to GLUT1-mediated glycolysis in HCC. These findings suggest that HCC patients with high levels of GLUT1 may benefit from icaritin administration. Besides, monitoring serum glycolystic biomarkers such as lactate dehydrogenases (LDH) and lactate can reflect patients’ response to icaritin.

According to previous studies, lncRNA FAM99A has been proven to be downregulated in many cells, like trophoblast cells (9). Moreover, in recent years, a few studies have explored the role of FAM99A in HCC cells and how FAM99A relates to HCC metastasis (11). Here, FAM99A was first identified as an upregulated lncRNA in icaritin treatment, indicating that FAM99A participates in the effect of icaritin in HCC. Moreover, we found that FAM99A has a negative correlation with the cell viability of HCC cells. We then confirmed that the knockdown of FAM99A could block the inhibition of HCC cell proliferation *via* a high concentration of icaritin. These data indicate that icaritin can affect the proliferation and the Warburg effect of HCC cells *via* regulating the expression of FAM99A. In this study, we uncovered the first lncRNA target of icaritin in HCC, which may help to identify icaritin-sensitive cases in clinical practice.

The JAK2/STAT3 pathway has been studied in recent years in relation to HCC cells and has been identified as a critical target of icaritin. Previous studies show that ANGPTL1 can regulate the JAK2/STAT3 pathway in HCC cells (30); also, lncRNA WT-AS1 can activate the JAK2/STAT3 pathway in HCC (31). Additionally, the JAK/STAT3 pathway’s involvement in the effects of icaritin in HCC has been demonstrated in a previous report (17). Our study was the first to explore the association between icaritin with FAM99A and the JAK2/STAT3 pathway in HCC. We revealed that FAM99A can inhibit the JAK2/STAT3 pathway by reducing the phosphorylation level of both JAK2 and STAT3. Furthermore, we discovered that FAM99A interacted with EIF4B to interfere with the EIF4B-mediated translation of gp130 and gp80 in HCC cells.

Previous studies have shown that SOCS3 inhibits the JAK2/STAT3 pathway in hepatocyte (32) and multiple myeloma cells (33). Similarly, our study was the first to verify that FAM99A can block the JAK2/STAT3 pathway *via* interacting with miR-299-5p and promoting SOCS3 in HCC cells. We further confirmed that FAM99A can inhibit STAT3 phosphorylation to affect the JAK2/STAT3 activity *via* binding to JAK2 at the JH1 catalytic domain. Finally, we established a xenograft model and confirmed that the overexpression of FAM99A inhibits HCC tumor growth, downregulates GLUT1, and blocks nuclear translocation of STAT3 *in vivo*.

Our findings also have clinical significance. Previous studies have demonstrated that glycolysis contributes to multiple cancer biology processes especially for immune escape and is involved in immune therapy resistance by secreting lactate and upregulating programmed death-ligand 1 expression (34, 35). Our study demonstrated that icaritin is a glycolysis inhibitor and thus has the potential to be administered as an immune enhancer in HCC patients treated with immunotherapy, such as anti-programmed cell death protein 1 therapy.

## Conclusion

**Figure 9:**
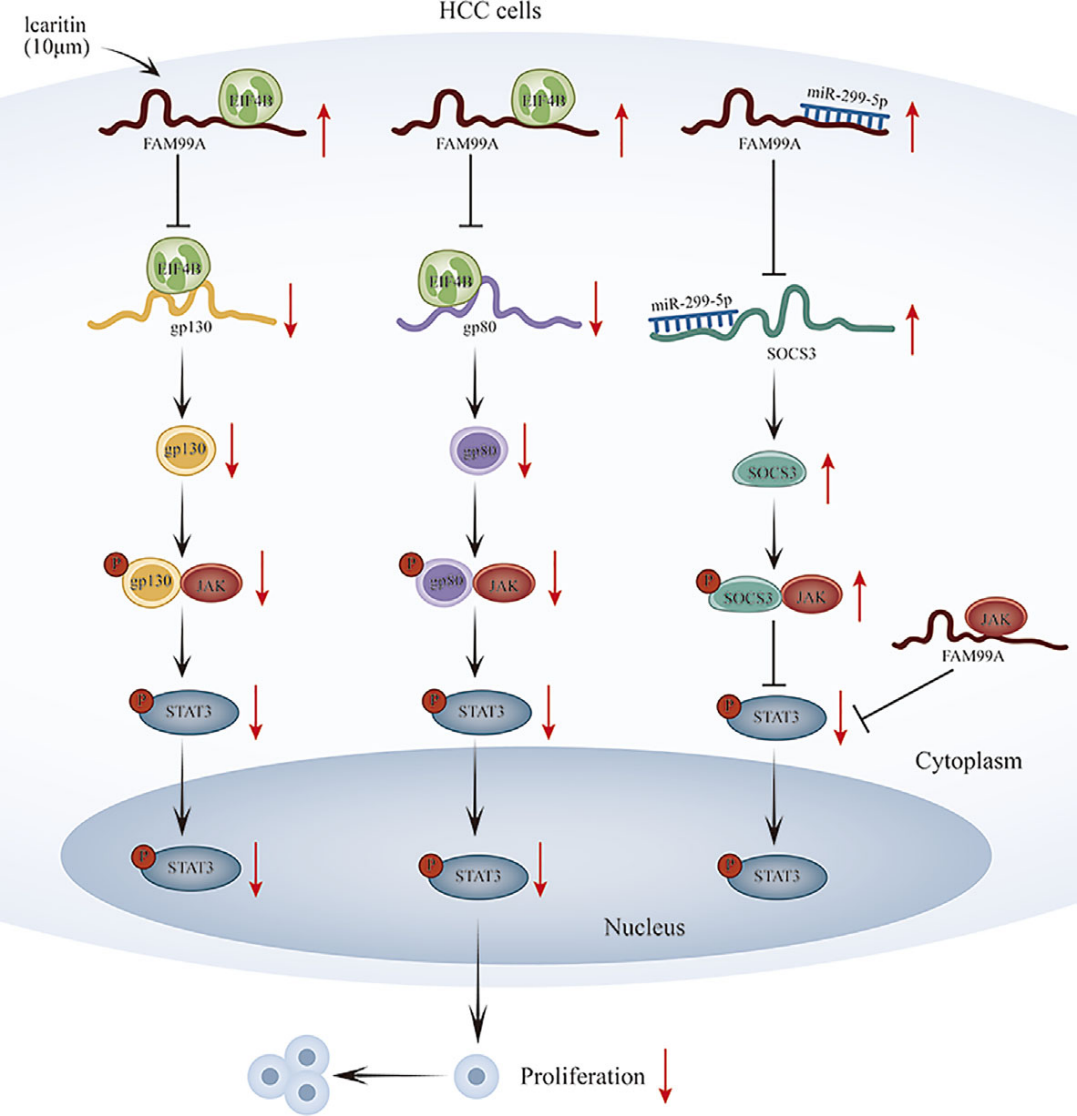
Schematic diagram of icaritin-induced FAM99A affects glycolysis via regulating the JAK2/STAT3 signal pathway in HCC.

Our novel finding is that icaritin-induced FAM99A can affect GLUT1-mediated glycolysis *via* regulating the JAK2/STAT3 pathway in HCC cells. In terms of mechanism, we revealed that FAM99A upregulates SOCS3 level through miR-299-5p and binds to EIF4B to block gp130 and gp80 translation to reduce p-STAT3 and block the JAK2/STAT3 pathway. However, further detailed studies are still needed to verify our findings. A graphical abstract has also been provided for reference. (**Figure 9**).

## Data Availability Statement

The original contributions presented in the study are included in the article/supplementary material. Further inquiries can be directed to the corresponding author.

## Ethics Statement

The animal study was reviewed and approved by the Ethics Committee of Nanjing University of Chinese Medicine (NO. ACU201103).

## Author Contributions

Conception and design: SQ. *In vitro* and *in vivo* experiments: XZ and YG. Analysis and interpretation of data: ZJ, AY, and ZY. Writing and review: XZ and SQ. All authors contributed to the article and approved the submitted version.

## Conflict of Interest

The authors declare that the research was conducted in the absence of any commercial or financial relationships that could be construed as a potential conflict of interest.

## Publisher’s Note

All claims expressed in this article are solely those of the authors and do not necessarily represent those of their affiliated organizations, or those of the publisher, the editors and the reviewers. Any product that may be evaluated in this article, or claim that may be made by its manufacturer, is not guaranteed or endorsed by the publisher.
